# Supplementary material for: Nf-Root: A Best-Practice Pipeline for Deep-Learning-Based Analysis of Apoplastic pH in Microscopy Images of Developmental Zones in Plant Root Tissue
Source: Quant Plant Biol. 2024 Dec 23;5:e12. doi: 10.1017/qpb.2024.11 (PMC11706687; doi:10.1017/qpb.2024.11)
Supplement: Wanner et al. supplementary material [file S2632882824000110sup001.zip › nf-root_Supplementary Material.docx]

# Supplementary Material


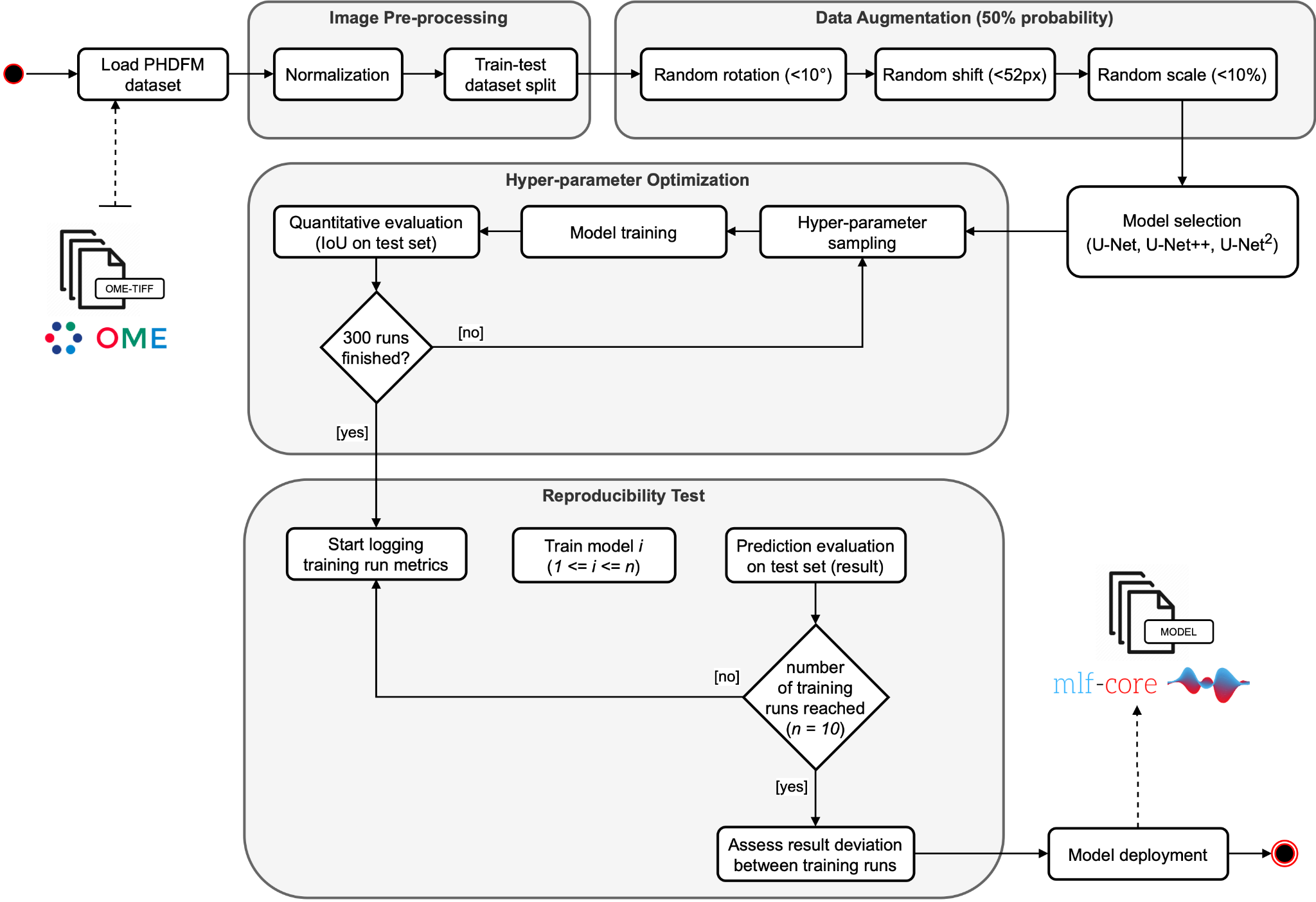


*Supplementary Figure S1. Overview of the model training, hyperparameter selection and deployment. Hyperparameter optimization was conducted over 300 runs with a Bayesian and hyperband optimization strategy. After normalization and splitting of the dataset, data augmentation is applied. Following hyperparameter optimization over 300 runs reproducibility is tested and the model can be deployed.*


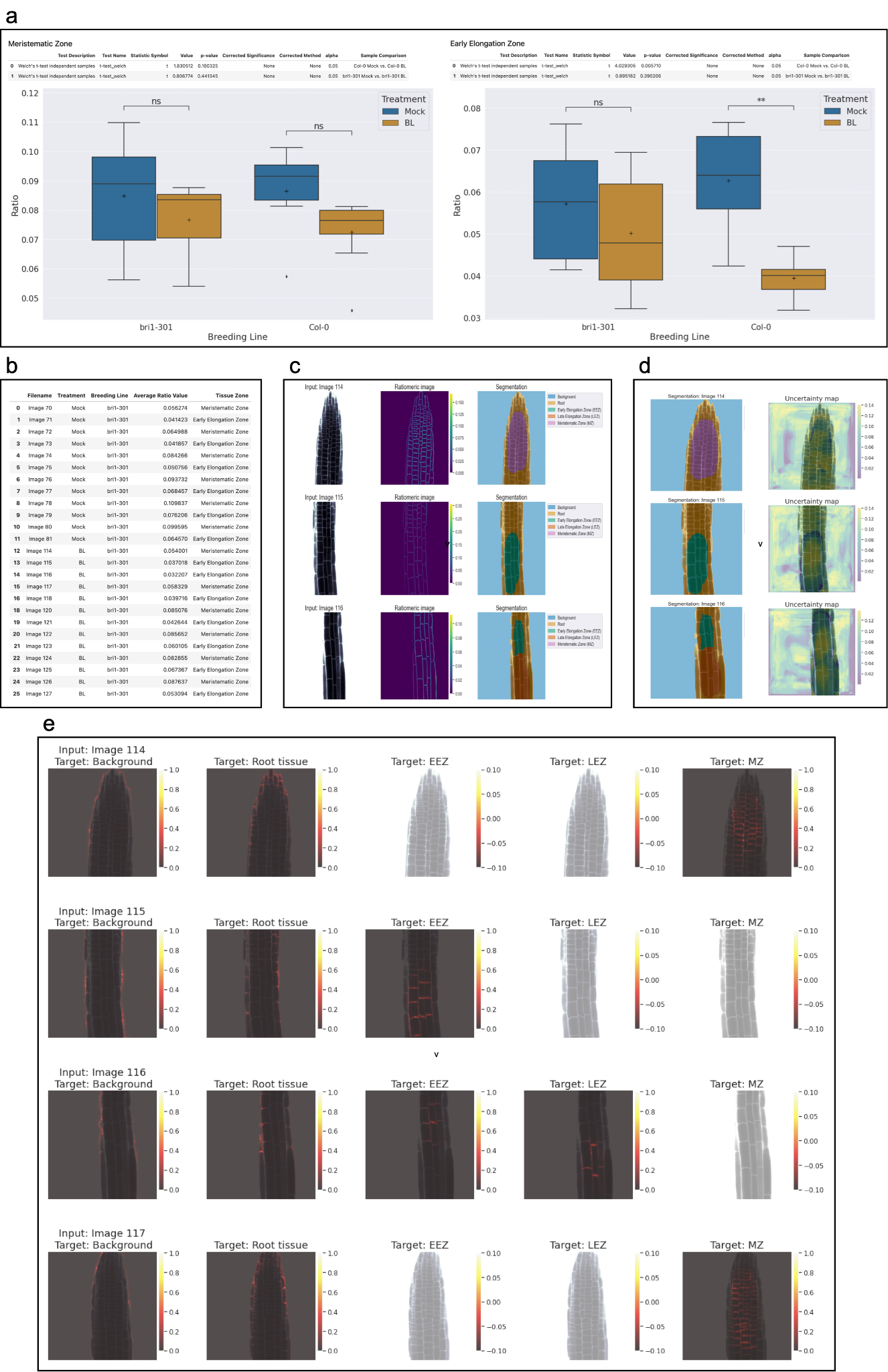


*Supplementary Figure S2. Visual overview of the pipeline report document, depicting different report sections. (a) View of the section for ratiomeric value statistics per tissue types. (b) View of the section that reports ratio average values for tissue zones and experimental factors. (c) Results section depicting input image, and the corresponding ratiomeric image and predicted segmentation mask, for all input images. (d) Results section showing uncertainty maps, for all input images. (e) Result section showing interpretability maps, for all input images and all target classes (segmentation classes).*

*
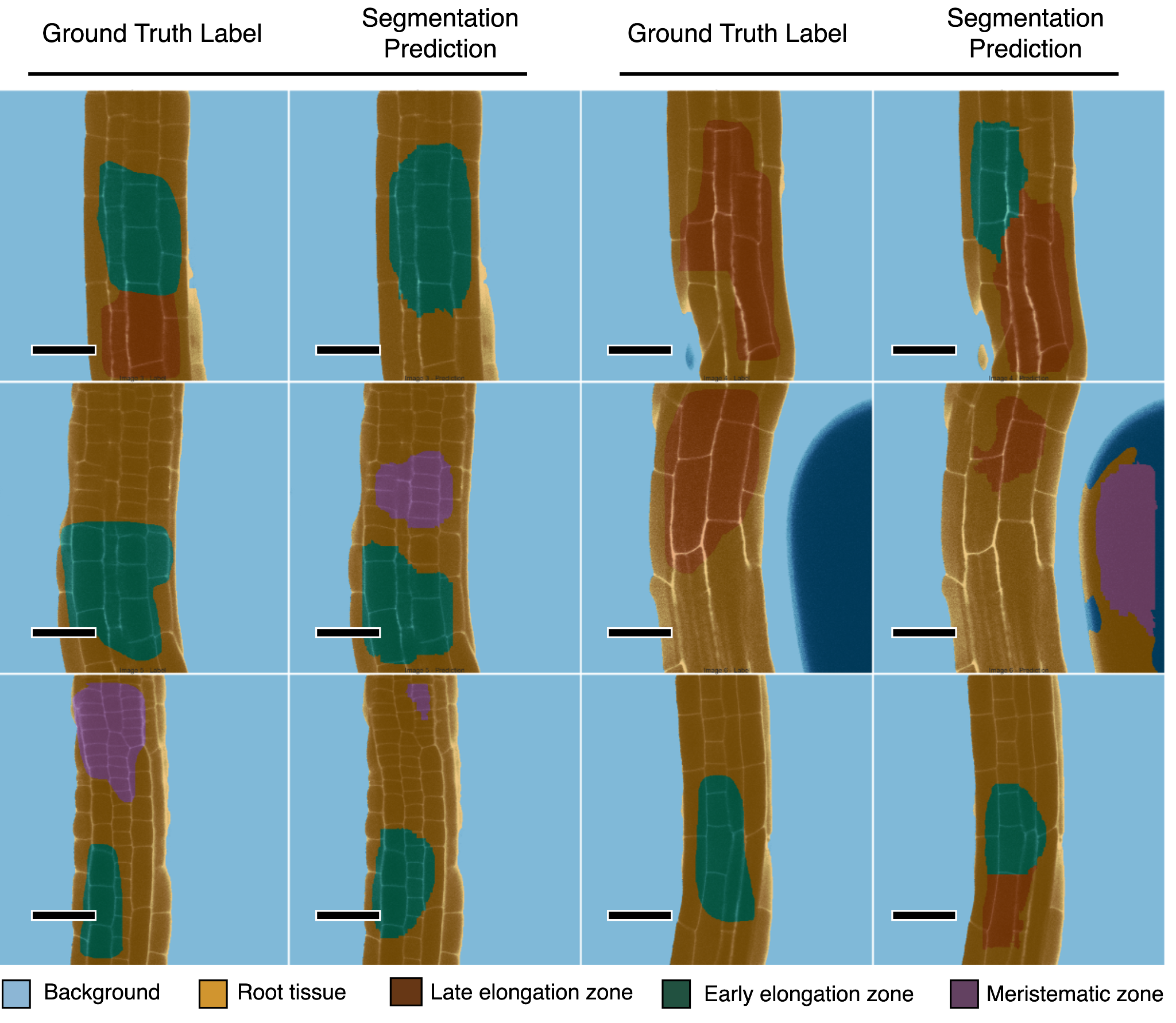
*

*Supplementary Figure S3. Examples of poor segmentation performance. Two columns of visual comparisons between tissue segmentation predictions and the corresponding ground truth, showing examples of images with poor segmentation performance. Scale bars = 53.14 µm.*

|  | Background | Root | EEZ | LEZ | MZ |
| --- | --- | --- | --- | --- | --- |
| Class frequency | 64.18 | 18.90 | 4.13 | 7.35 | 5.45 |
| Annotated Images | 601 | 601 | 215 | 233 | 212 |

*Supplementary Table S4. Pixel distributions of all five classes (Background, Root tissue, EEZ. LEZ, MZ) in the PHDFM dataset.*

| Model | IoU background | IoU root | IoU  EEZ | IoU LEZ | IoU  MZ | IoU  average | Model parameters |
| --- | --- | --- | --- | --- | --- | --- | --- |
| U-Net | 0.99 | 0.70 | 0.52 | 0.66 | **0.76** | 0.72 | 7.8 M |
| U-Net++ | 0.99 | 0.69 | 0.54 | 0.69 | 0.72 | 0.73 | 9.006 M |
| U-Net^2 | 0.99 | **0.72** | **0.60** | **0.71** | 0.73 | **0.75** | 44.040 M |

*Supplementary Table S5. Model evaluation using the Jaccard index, also referred as Intersection over Union (IoU). Three models were evaluated, based on the U-Net architecture. The performance metric IoU is reported for all five segmentation classes. This table reports the number of trainable parameters (weights and biases) for each of the models, in terms of millions (M) of parameters.*

| Filename | Treatment | Plant line |
| --- | --- | --- |
| Image 1 | Mock/Water | bri1-301 |
| Image 2 | Brassinolide | bri1-301 |

*Supplementary Table S6. Exemplary metadata input table, for two different images of bri1-301 mutants, one treated with water, the other with Brassinolide.*
