# Supplementary figures and images for: Nf-Root: A Best-Practice Pipeline for Deep-Learning-Based Analysis of Apoplastic pH in Microscopy Images of Developmental Zones in Plant Root Tissue
Source: Quant Plant Biol. 2024 Dec 23;5:e12. doi: 10.1017/qpb.2024.11 (PMC11706687; doi:10.1017/qpb.2024.11)

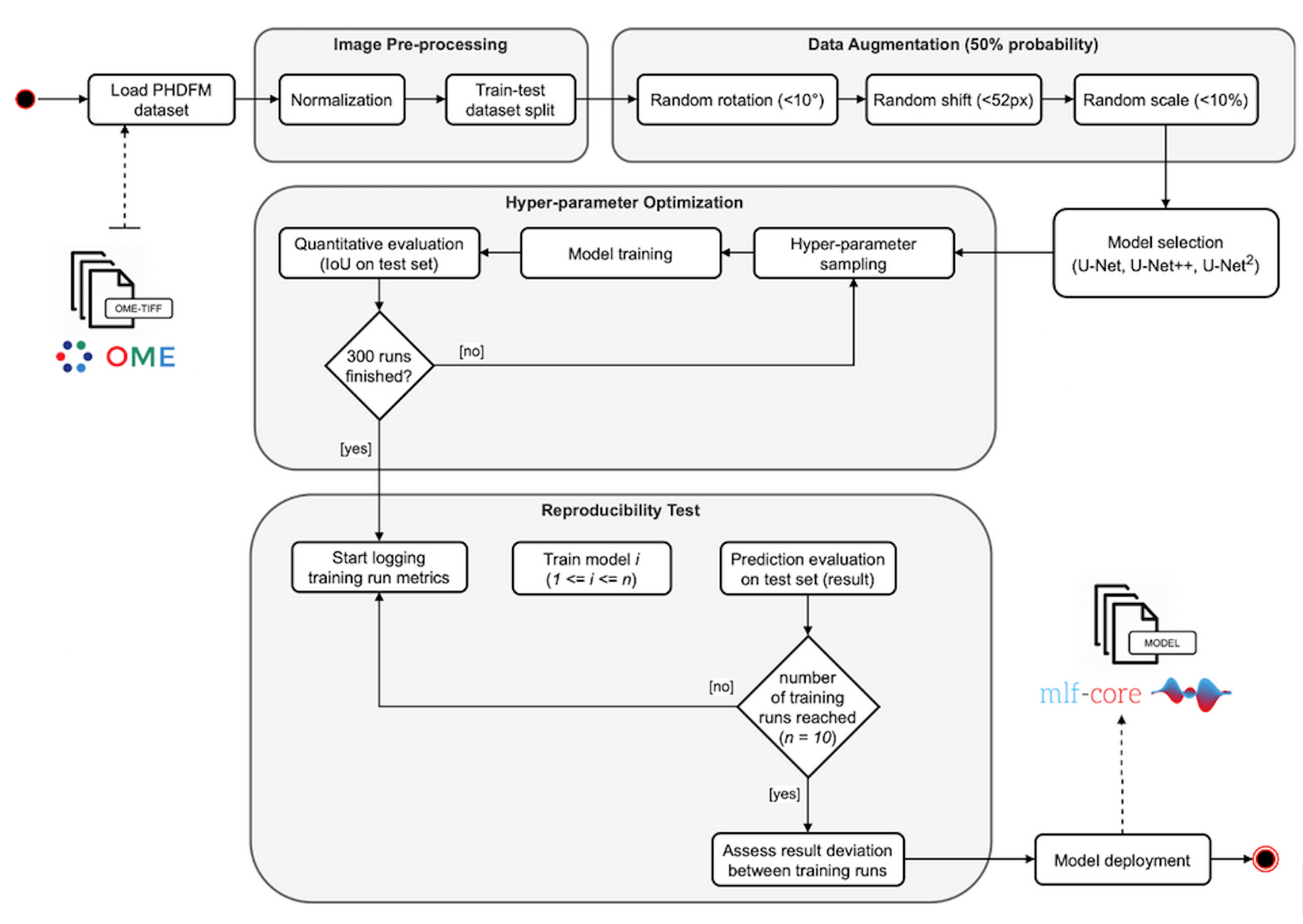

Supplement: Wanner et al. supplementary material [file S2632882824000110sup001.zip › nf-root_fig_S1.png]

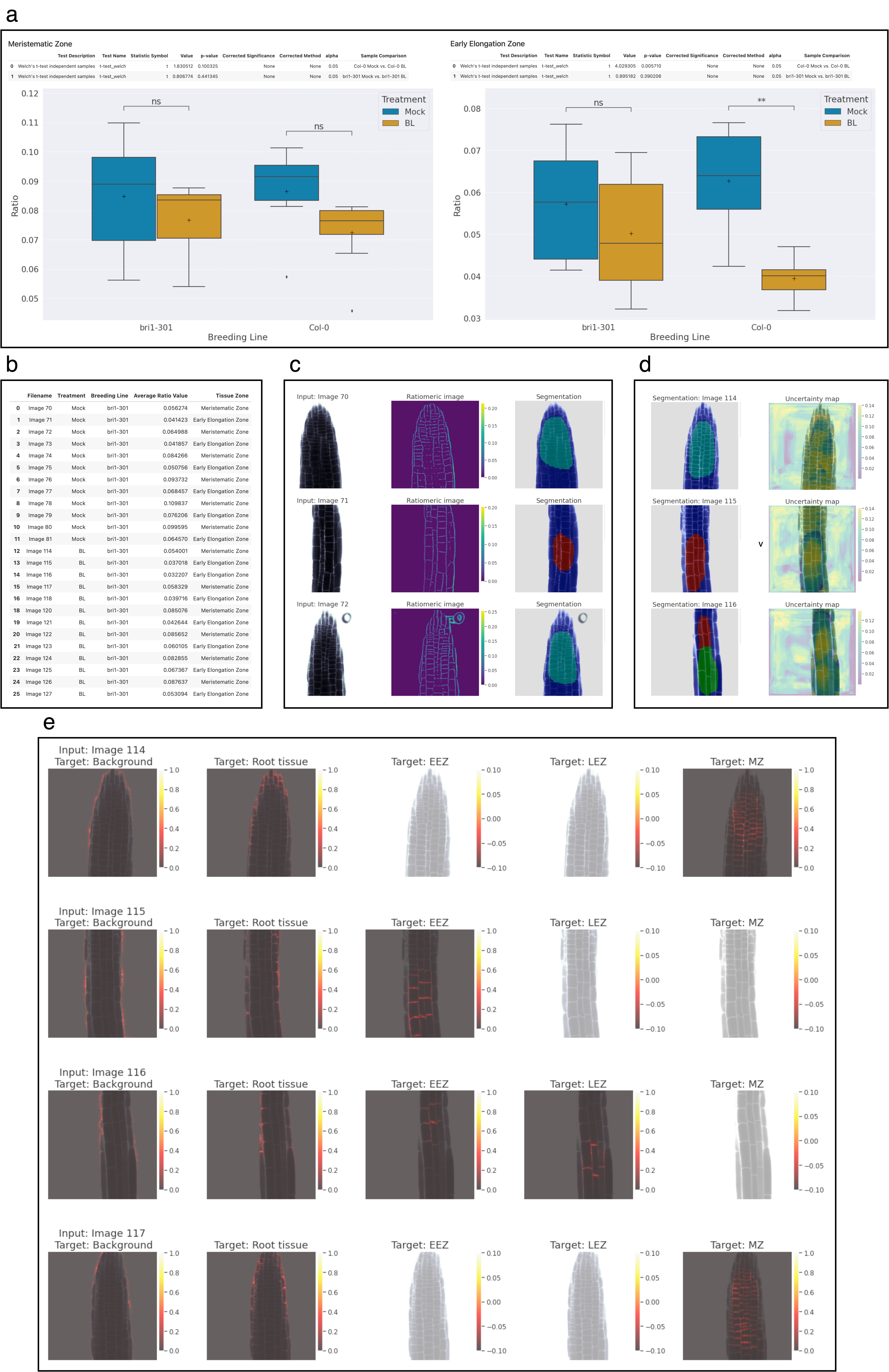

Supplement: Wanner et al. supplementary material [file S2632882824000110sup001.zip › nf-root_fig_S2.png]

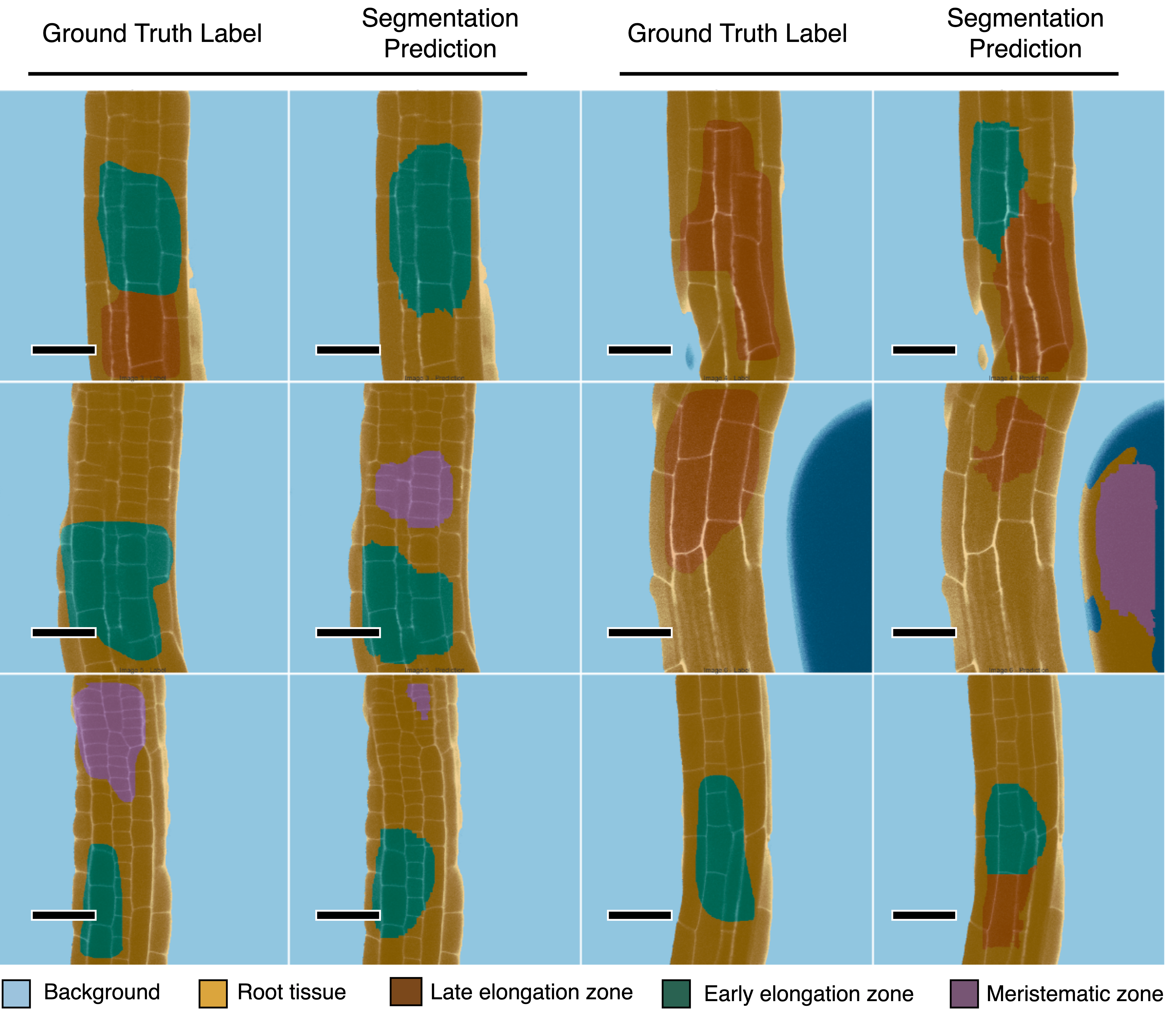

Supplement: Wanner et al. supplementary material [file S2632882824000110sup001.zip › nf-root_fig_S3.png]
